# Mismatch Repair-Deficient FIGO Stage IA Endometrial Adenocarcinoma With Negative Nodal Biopsy Presenting as Isolated Orbital Metastasis

**DOI:** 10.7759/cureus.83216

**Published:** 2025-04-29

**Authors:** Steven B Barker, Aditya Ghosh, Christie Taylor, Shravanti Macherla

**Affiliations:** 1 Internal Medicine, Northeast Georgia Medical Center Gainsville, Gainesville, USA; 2 Internal Medicine, Mayo Clinic, Rochester, USA; 3 Internal Medicine, Augusta University - Medical College of Georgia, Augusta, USA; 4 Hematology and Oncology, Longstreet Cancer Center, Gainesville, USA

**Keywords:** early-stage endometrial cancer, endometrial adenocarcinoma, international federation of gynaecology and obstetrics (figo) staging, lymph node-negative, microsatellite instability, mismatch repair deficiency, orbital metastases, pelvic lymphadenectomy

## Abstract

Endometrial cancer is the most common malignancy of the female genital tract in the United States and the fourth most common cancer among women worldwide. Recently, the pathological classification of endometrial cancer has expanded from a two-tiered system to a four-group system based on molecular profiling. Typically, metastatic endometrial cancer spreads via the lymphatic system, most frequently to the retroperitoneum. Hematogenous spread is rare but can occur. We present a case of an 82-year-old female with a history of FIGO (International Federation of Gynecology and Obstetrics) stage IA endometrial adenocarcinoma, status post total hysterectomy and bilateral salpingo-oophorectomy, with negative lymph node biopsy, who later presented with eye swelling and pain. Imaging revealed metastatic disease to the orbit with invasion of the orbital roof. She subsequently underwent surgical resection, radiation, and systemic chemotherapy. Recurrence of grade IA endometrial carcinoma following hysterectomy and bilateral salpingo-oophorectomy with negative lymph node biopsies is exceedingly rare. The risk of pelvic lymph node involvement in stage IA disease is ≤3%, with a five-year progression-free survival rate of 95-98%. Current methods of metastatic assessment include lymphadenectomy and sentinel lymph node biopsy, although the optimal approach remains debated. This case underscores the need for continued advancements in risk stratification and early identification of patients with occult metastatic disease.

## Introduction

Endometrial cancer is the most common malignancy of the female genital tract in the United States and the fourth most common cancer among women worldwide [1,2]. Although the average age at diagnosis is 63 years, approximately 20-25% of cases occur in premenopausal women [3-6].

Historically, endometrial carcinoma has been classified into two types, Type I and Type II, based on histopathological characteristics [7-10]. However, the Cancer Genome Atlas (TCGA) has proposed a more refined molecular classification comprising four subgroups: POLE-ultramutated, microsatellite instability-high (MSI-H), copy-number high (usually associated with TP53 mutations), and copy-number low (no defining molecular alterations) [11]. This classification is increasingly used to guide prognosis and personalize treatment strategies.

The most prominent clinical features of endometrial carcinoma include abnormal uterine bleeding, intermenstrual bleeding, irregularly heavy bleeding, or any postmenopausal bleeding [6,9]. Regional extension of endometrial carcinoma may present as pelvic pain, pressure, or mass; cervical abnormalities; abdominal distension; and changes in bowel or bladder function [6,12]. Metastatic disease usually spreads lymphatically (most commonly to the retroperitoneal space), but it can rarely spread hematogenously (most commonly to the lungs). However, it is essential to note that pelvic examination and laboratory results are typically normal in patients with endometrial carcinoma [6,9,12].

The patient in this report presented with Type I endometrial cancer with microsatellite instability (MSI), indicating a defective mismatch repair system. Metastasis of endometrial adenocarcinoma is extremely rare, and the true incidence of orbital metastasis of endometrial adenocarcinoma is unknown. To date, orbital metastasis of endometrial adenocarcinoma has only been reported in case reports.

## Case presentation

An 82-year-old woman (G4P4, menarche at age 15, menopause at age 52) with a history of FIGO (International Federation of Gynecology and Obstetrics) stage IA (cT1a, cN0, cM0) endometrial adenocarcinoma presented with progressive left eye pain, proptosis, and swelling approximately 19 months after her initial cancer diagnosis. Her family history was notable for ovarian cancer in her mother, thyroid cancer in her sister, and breast cancer in her daughter.

**Figure 1:**
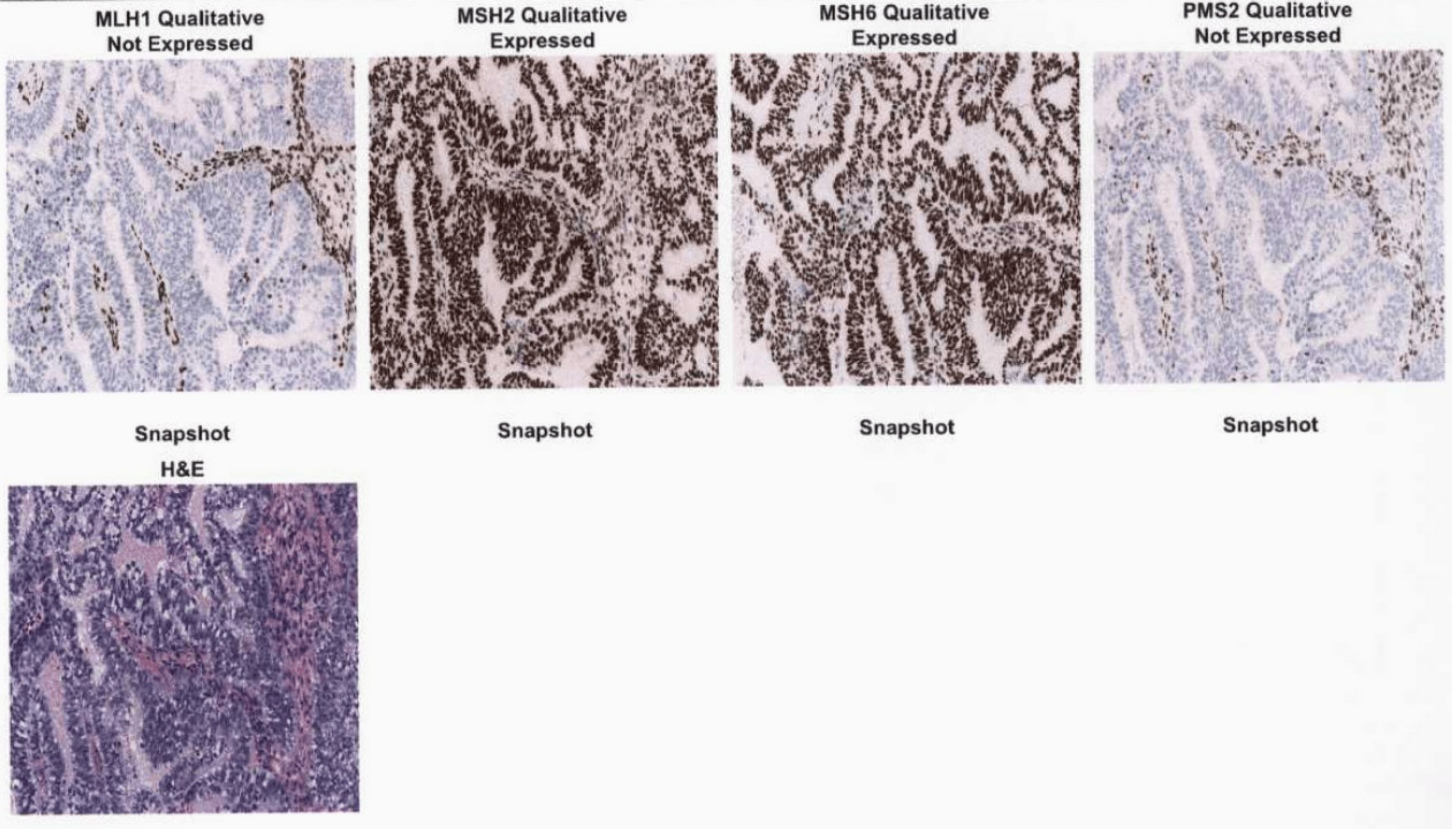
Mismatch repair enzyme staining following dilation and curettage (D&C). Images viewed at 100× magnification.

Initially, at age 80, she presented to her primary care physician with abnormal uterine bleeding. She was referred to a gynecologist and underwent hysteroscopy with dilation and curettage. Two polyps and a mass-like structure were identified and biopsied. Pathology revealed endometrioid adenocarcinoma with absent or low expression of mismatch repair (MMR) proteins MLH1 and PMS2, while MSH2 and MSH6 expression was retained (Figure 1).

Genomic testing demonstrated MLH1 promoter hypermethylation (53.5%), and microsatellite instability testing confirmed MSI-high status. The patient subsequently underwent total hysterectomy and bilateral salpingo-oophorectomy with pelvic washings. Cytological analysis and lymphadenectomy of eight right pelvic lymph nodes and seven left pelvic lymph nodes revealed no malignancy. Final pathology confirmed FIGO stage IA endometrioid adenocarcinoma (Müllerian primary) without myometrial invasion or angiolymphatic involvement of the fallopian tubes or ovaries. Given the absence of lymphatic spread, adjuvant radiation or chemotherapy was not recommended.

**Figure 2:**
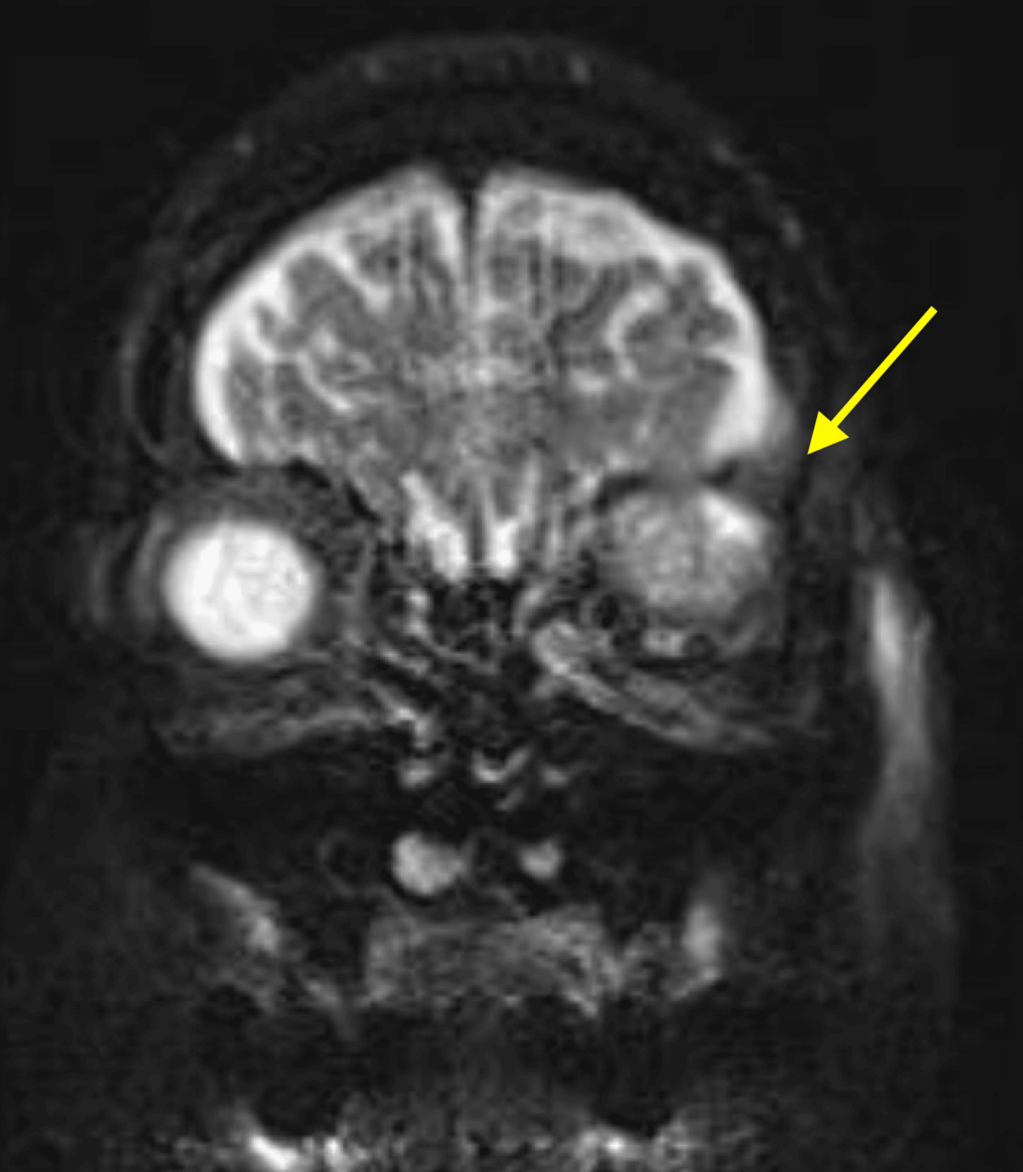
Orbital MRI with and without intravenous contrast showing a large, avidly enhancing, solid and cystic mass in the left superior orbit with intracranial extension through the left orbital roof. There is direct dural involvement and mass effect on the left inferior frontal lobe. The yellow arrow denotes the left orbital mass, which displaces the globe and invades the left orbital roof.

Nineteen months later, the patient developed left upper eyelid swelling and visual disturbances. A PET/CT was scheduled, but the patient presented to the emergency department shortly thereafter with worsening proptosis and vision loss. She was started on oral corticosteroids and empiric antibiotics, and urgent ophthalmology and neurosurgery consultations were obtained. An MRI of the orbit revealed a 4.0 × 3.2 × 3.1 cm mass in the left orbit with associated proptosis and inferior displacement of the left globe (Figure 2). The patient subsequently underwent a multidisciplinary surgical procedure, including a left orbitozygomatic craniotomy with stereotactic volumetric tumor resection, complex orbital roof and rim reconstruction, and exploratory orbitotomy with biopsy and mass resection.

Postoperative histopathology confirmed metastatic adenocarcinoma consistent with a Müllerian primary, with loss of MLH1 and PMS2 expression identical to the patient’s original tumor profile. She received postoperative adjuvant orbital radiation therapy. An orbitotomy with cytologic washings and biopsy was performed. Flow cytometry of the washings was negative for definitive malignancy; however, biopsy demonstrated poorly differentiated carcinoma with both acute and chronic inflammation. "Immunohistochemical staining was positive for Super K, cytokeratin 7 (CK7), progesterone receptor (PR), and estrogen receptor (ER), suggestive of metastatic Müllerian carcinoma.

**Figure 3:**
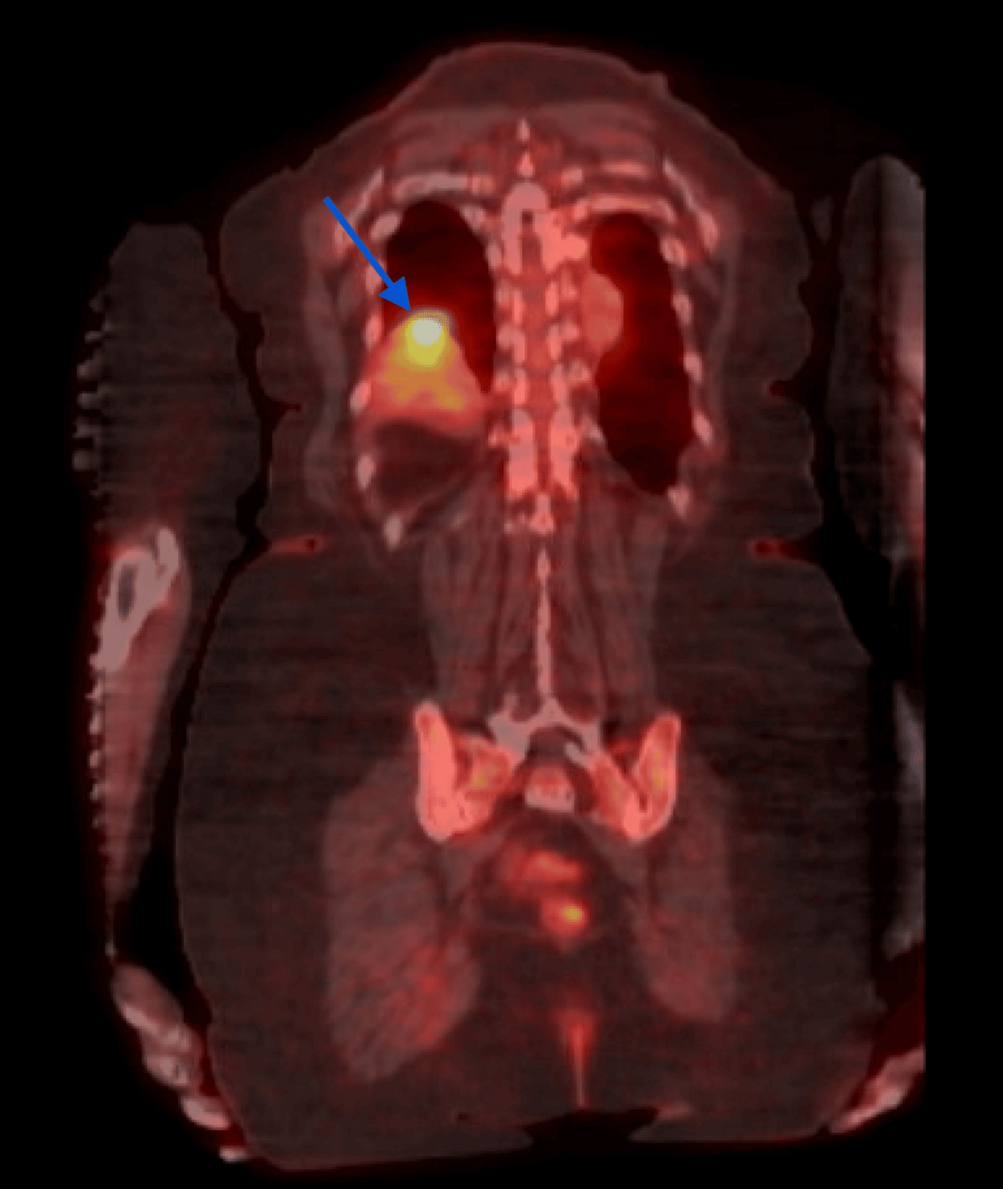
Whole-body PET/CT revealing a metastatic lung nodule. The blue arrow indicates metastatic lung nodule.

**Figure 4:**
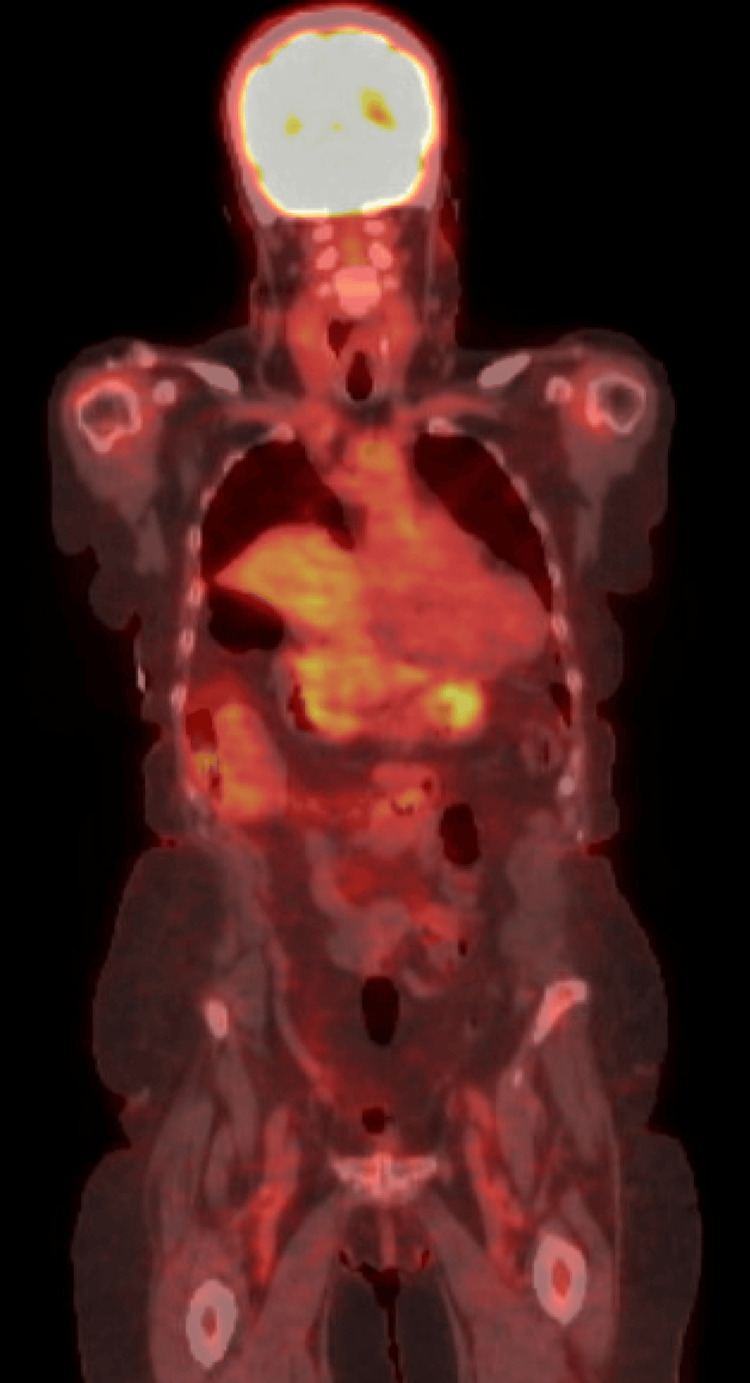
Whole-body PET/CT revealing no residual or metastatic disease in the abdomen or pelvis.

A whole-body PET/CT scan revealed a 1.9 cm pulmonary nodule in the posterior aspect of the right lower lobe (Figures 3, 4). A CT-guided biopsy of the right lower lobe pulmonary nodule confirmed metastatic, poorly differentiated adenocarcinoma. Approximately 1.5 months after discharge, she began radiation therapy. She remained on oral corticosteroids to manage periorbital and intracranial edema. Pembrolizumab 200 mg every three weeks was initiated two months post-discharge, and she continues to receive Pembrolizumab every three weeks since diagnosis.

## Discussion

Endometrial carcinoma is primarily a histopathologic diagnosis. In this case, the tumor was classified as FIGO stage IA, indicating superficial or no myometrial invasion. First-line management for stage IA disease typically involves surgical resection, total hysterectomy with bilateral salpingo-oophorectomy, without adjuvant therapy, as recurrence is uncommon. The risk of pelvic lymph node involvement in stage IA endometrial cancer is estimated at ≤3%, and the five-year progression-free survival rate ranges from 95% to 98% [6-11]. However, identifying the small subset of patients at higher risk for recurrence or distant metastasis remains a clinical challenge.

The role of lymph node assessment continues to be an area of active debate. While some surgeons perform systematic lymphadenectomy for staging purposes, others favor a more conservative approach, especially in low-risk disease. Sentinel lymph node (SLN) biopsy has emerged as a viable alternative, offering adequate staging with lower surgical morbidity. Advocates for limited surgery argue that the likelihood of nodal metastasis in early-stage disease is low and that lymphadenectomy provides no therapeutic benefit in the absence of nodal involvement. Additionally, omitting lymphadenectomy reduces operative time, lowers complication rates, and shortens recovery. However, this approach may lead to underdiagnosis of occult metastatic disease, delayed initiation of systemic therapy, and worse outcomes in select patients [6].

Several risk factors have been proposed to guide decision-making around lymph node dissection, including patient age, tumor grade, depth of myometrial invasion, lymphovascular space invasion (LVSI), and histologic subtype [13,14]. Biomarkers such as CA-125 have also been investigated for their potential to predict extrauterine spread [15,16]. Molecular profiling is increasingly used to stratify risk and guide treatment decisions, particularly in ambiguous or high-risk cases.

The TCGA molecular classification provides additional insight into tumor biology. In particular, MMR-D or MSI-H tumors, as seen in this patient, are associated with unique therapeutic implications. Notably, MSI-H tumors tend to respond poorly to conventional chemotherapy but demonstrate improved outcomes with immunotherapy, particularly immune checkpoint inhibitors such as pembrolizumab [17,18]. Despite promising data, standardized treatment protocols based on molecular classification have yet to be universally adopted.

This case highlights the importance of integrating molecular profiling into routine diagnostic and treatment workflows. Although the patient had low-stage, node-negative disease, her MSI-H profile and eventual hematogenous spread underscore the limitations of conventional staging and the need for personalized treatment strategies. Early identification of high-risk molecular subtypes may help guide the use of adjuvant therapies and prompt closer surveillance in patients with otherwise low-risk histology.

## Conclusions

Although FIGO stage IA endometrial adenocarcinoma is generally associated with a favorable prognosis, this case illustrates how mismatch repair deficiency can significantly alter the disease trajectory. The unexpected development of distant metastasis in a patient with negative lymph node biopsies underscores the limitations of traditional staging and highlights the need for molecular profiling in early disease. A tailored approach to surveillance and treatment, particularly in patients with MSI-H tumors, may improve long-term outcomes.
